# The effect of myo‐inositol supplementation on AMPK/PI3K/AKT pathway and insulin resistance in patients with NAFLD

**DOI:** 10.1002/fsn3.4267

**Published:** 2024-07-16

**Authors:** Taha Aghajani, Sara Arefhosseini, Mehrangiz Ebrahimi‐Mameghani, Reza Safaralizadeh

**Affiliations:** ^1^ Department of Animal Biology, Faculty of Natural Sciences University of Tabriz Tabriz Iran; ^2^ Student Research Committee Tabriz University of Medical Sciences Tabriz Iran; ^3^ Nutrition Research Center, Department of Biochemistry and Diet Therapy, Faculty of Nutrition and Food Sciences Tabriz University of Medical Sciences Tabriz Iran

**Keywords:** gene expression, insulin resistance, myo‐inositol, non‐alcoholic fatty liver disease

## Abstract

Insulin resistance (IR) is the pivotal pathological hit in non‐alcoholic fatty liver disease (NAFLD). There is specific attention to combination/conjugated therapies for NAFLD management. As myo‐inositol (MI) has been shown to improve IR in animal and human trials, this study aimed to investigate the influence of MI supplementation on glycemic response and IR through AMPK/PI3K/AKT signaling pathway in obese patients with NAFLD. This double‐blinded placebo‐controlled randomized clinical trial was conducted on 48 obese (BMI = 30–40 kg/m^2^) patients with NAFLD who were randomly assigned to receiving either MI (4 g/day) or placebo (maltodextrin 4 g/day) group for 8 weeks. Before and after the trial, weight, height, serum glycemic parameters (inc. fasting glucose and insulin) as well as IR indices were assessed. Moreover, the mRNA expression levels of AMPK, AKT, and PDK‐1 in peripheral blood mononuclear cells (PBMCs) were determined. MI supplementation resulted in significant increases in the fold changes of AMPK, AKT, and PDK‐1 genes (*p* = .019, *p* = .049, and *p* = .029, respectively). Indeed, IR improved in terms of all IR indices in MI group (*p* < .05) after adjusting for the confounders, apart from quantitative insulin sensitivity check index (QUICKI). The results showed that MI supplementation not only upregulated AMPK, AKT, and PDK‐1 mRNA in PBMCs but also improved IR in obese patients with NAFLD.

## Graphical abstract

This double‐blinded placebo‐controlled randomized clinical trial on obese patients with NAFLD showed that myo‐inositol (MI) supplementation (4 g/day) for 8 weeks resulted in significant increases in the fold changes of AMPK, AKT, and PDK‐1 genes in peripheral blood mononuclear cells. MI as a molecule with insulin‐mimetic features improved insulin resistance (IR) in terms of all IR‐related indices, apart from quantitative insulin sensitivity check index. Hence, MI supplementation could favorably improve glucose metabolism‐related genes and IR in patients with NAFLD.

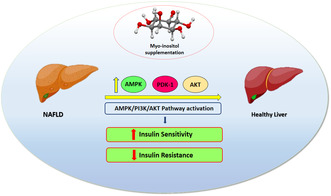

## 1 INTRODUCTION

Non‐alcoholic fatty liver disease (NAFLD) as a complex multisystem disease is diagnosed by over‐accumulation of lipid in the liver, followed by a range of intra/extra‐hepatic and metabolic complications and considered the most common cause for liver transplantation (Byrne & Targher, 2015; Lonardo et al., 2019). The worldwide burden of NAFLD has been tremendously increased from 25% to 38% over the last 30 years (Younossi et al., 2023). The relationship between NAFLD and insulin resistance (IR)‐related conditions such as type 2 diabetes mellitus (T2DM), metabolic syndrome (Mets) and even, polycystic ovary syndrome (PCOS) has been proposed (Arefhosseini et al., 2022; Byrne & Targher, 2015). A complex of hereditary, metabolic, and environmental factors leads to NAFLD progression (Rong et al., 2022). The “Multi‐hit” theory postulates the interaction among genetical susceptibility, gut dysbiosis, oxidative stress, chronic inflammation, and IR as the prominent step in the development of NAFLD (Buzzetti et al., 2016). IR stimulates adipocyte lipolysis and suppresses myocyte glycogenesis, which in turn triggers the influx of fatty substrates and glucose to the liver. Moreover, hyperinsulinemia promotes de novo lipogenesis and facilitates hepatic fat deposition (Song et al., 2023).

A number of cellular signaling pathways mediate IR and thereby are associated with the pathogenesis of NAFLD (Savova et al., 2023). AMP‐activated protein kinase/phosphoinositol‐3‐kinase/protein kinase‐B (AMPK/PI3K/AKT) signaling pathway is implemented in the regulation of a plethora of proteins involved in metabolic homeostasis, particularly in insulin transduction (Savova et al., 2023; Zhou et al., 2023). Therefore, targeting AMPK/PI3K/AKT pathway appears to be a novel treatment strategy for IR‐related conditions such as NAFLD. The current body of evidence suggests lifestyle modifications and weight reduction as the first‐line treatments for NAFLD; meanwhile, nutraceuticals such as inositols (INS) have been recently gained great attention (Arefhosseini, Roshanravan, Tutunchi, et al., 2023; Rooholahzadegan et al., 2023).

Myo‐inositol (MI)—a member of cyclo‐hexane polyols and also a water‐soluble sugar alcohol—has been introduced with outstanding insulin‐mimetic features (Dinicola et al., 2017). MI is found in all living cells and human diet, particularly in fruits, seeds, nuts, and grains (DiNicolantonio & O'Keefe, 2022). Various cellular functions have been attributed to MI, ranging from membrane biogenesis and energy homeostasis to hormonal signaling transduction (Antonowski et al., 2019; Arefhosseini, Roshanravan, Tutunchi, et al., 2023). Current evidence suggests that MI is a drug of choice in the management of IR‐related complications (DiNicolantonio & O'Keefe, 2022; Showell et al., 2018) through its ability as an insulin‐mimetic agent in increasing insulin sensitivity, cellular glucose uptake and improving glucose disturbances by the activation of PI3K/AKT pathway (Özturan et al., 2019). Pani et al. (2020) documented the positive effects of MI in improving hepatic lipid accumulation and liver enzymes in animal models of NAFLD. Moreover, we have previously reported the favorable influences of 8‐week MI supplementation (4 g/day) plus nutritional recommendations on cardiovascular biomarkers, obesity indices, inflammation, and hepatic markers in obese patients with NAFLD (Arefhosseini, Roshanravan, Asghari, et al., 2023; Arefhosseini, Roshanravan, Tutunchi, et al., 2023).

To our knowledge, studies examining the effects of MI on IR in patients with NAFLD are limited. Moreover, the exact hypoglycemic mechanism of MI on IR and related genes are still unclear. Therefore, this study purposed to examine the effects of MI supplementation on IR and AMPK/PI3K/AKT signaling pathway in patients with NAFLD.

## 2 MATERIALS AND METHODS

### 2.1 Study procedures and population

The current double‐blind placebo‐controlled randomized clinical trial was conducted on newly diagnosed obese patients with NAFLD. The participants of the current study were patients aged 18 to 55 years with BMI ranged between 30 and 40 kg/m^2^ and suffering from mild to moderate liver steatosis. After the detection of liver steatosis by a single sonographist at fasting state through ultrasonography (Sonoace X4 Medisio, South Korea), and classification into mild, moderate, and severe degrees according to Hamaguchi criteria (Hamaguchi et al., 2007), NAFLD was confirmed by a gastroenterologist. Pregnant, lactating or menopause women, smokers or alcohol drinkers, those with a history of particular diets or using herbal/synthetic drugs as well as nutritional supplements during the last 3 months were excluded from the study. Patients taking any type of medications affecting lipid and glucose metabolism, contraceptives, and supplements were not included in this study. Moreover, co‐morbidity with, metabolic, gastro‐intestinal, and other liver disorders were also among exclusion criteria.

Subjects were outpatients recruited from the clinics affiliated with Tabriz University of Medical Sciences, Tabriz, Iran. In the current study, the protocols were approved by the Ethics Committee of Research vice‐chancellor of Tabriz University of Medical Sciences (TBZMED. REC.1400.567), and an informed consent form was obtained from all of the participants after explaining the study aims and procedures. This RCT was also registered at the Iranian Registry of Clinical Trials available at www.irct.ir (IRCT20100209003320N22).

As the current study was parallel with other ongoing RCTs (Arefhosseini, Roshanravan, Asghari, et al., 2023; Arefhosseini, Roshanravan, Tutunchi, et al., 2023), the sample size was mutually estimated based on Lee et al. (2019) study which reported the mean (standard deviation (SD)) change of serum triglyceride (TG) levels among patients with NAFLD who received d‐pinitol. We considered an effect size of 10 mg/dL of TG changes, confidence level, and power equal to 95% and 80% using sample size software (PASS; NCSS, LLC, US). The sample size was calculated as 18 for each group which increased to 22 by supposing 30% dropout rate.

The patients were randomly divided into either MI group or placebo group (1:1) by a research assistant not otherwise involved in the study and by Random Allocation Software (RAS) and randomized block procedure. The randomized block procedure of size 3 was considered follows: [gender (male vs. female), age (18–35 years vs. 36–55 years), and BMI (<35 kg/m^2^ vs. ≥35 kg/m^2^)].

Three‐digit code was used by the research assistant (entirely unrelated to the RCT) to prepare and package the supplement and placebo sachets for each of the groups. The allocation was blinded for the patients, assessors, and researcher till the end of the trial and after statistical analysis. MI powder was packaged in two‐gram sachets provided by Wholesale Health Connection in China. Those in the MI group (*N* = 25) were asked to consume 2 g of MI sachets, while the subjects in the placebo group (*N* = 26) received 2 g of maltodextrin before lunch and dinner for 8 weeks. The size, color, and other characteristics of the MI and placebo sachets were exactly similar. Unused sachets were asked to deliver every 2 weeks for assessing the compliance rate. For all patients, a healthy diet recommendation was also given, and weight was assessed fortnightly (Evert et al., 2014). All patients were also asked to continue their routine lifestyle and only adhere to the dietary guidelines.

Weight and height were assessed using stadiometer (Seca, Hamburg, Germany) with low clothes and no shoes by precision of 100 g and 0.5 cm, respectively, and body mass index (BMI) was calculated as weight (kg) divided by height squared (m^2^). As possible confounders, physical activity level and energy intakes were also determined at the baseline and at the end of the study. Physical activity level was estimated using the international physical activity questionnaire–short form (IPAQ‐SF) by reporting the amount of time spent engaging in each of the activities with varying intensities during the course of the previous week by the patients, and then metabolic equivalent of task (MET‐hours/week) scores was calculated (Committee IR, 2005). Moreover, daily energy intake was assessed using a 3‐day food recall (two non‐consecutive weekdays and a weekend). The average 3‐day food consumption was analyzed using Nutritionist IV software (First Databank, San Bruno, CA, USA), and energy intake was reported as kcal/day.

### 2.2 Molecular assay

In a fasting state, five ml of fresh peripheral blood was taken pre‐ and post‐intervention and collected in EDTA tubes (Vacutainer K2E). PBMCs were isolated using Ficoll‐Histopaque (Ficoll‐Paque, GmbH, Germany) density gradient centrifugation. The extraction and purification of total RNA were performed using Ambion Trizol LS reagent (Thermo Fisher Scientific, USA), based on the protocol of the manufacturer. RNA quantification and qualification were determined by NanoDrop spectrophotometer (NanoDrop One/One^c^, Thermo Scientific) and gel electrophoresis method, respectively. The relative sample OD at 260/280 and 260/230 was identified in order to check for any kind of contamination with DNA or protein. Then, RNA samples of study participants were frozen at −80°C until the subsequent procedures. Total RNA was reverse transcripted to complementary DNA (cDNA) library through polymerase chain reaction (PCR) via Moloney murine leukemia virus (MMLV), random hexamer primer, RNase inhibitor, dNTP mix, and cDNA synthesis buffer (BioFact™ RTase, South Korea). Subsequently, β‐actin gene was considered endogenous control in the normalization of target gene expression. Specific primer sequences for β‐actin, AMPK, AKT, and phosphoinositide‐dependent kinase‐1 (PDK‐1) genes were designed using Oligo v7.56 software (Bioneer Co. Chungwon, South Korea) and NCBI (Table 1). mRNA expression levels of AMPK, AKT, and PDK‐1 were evaluated through the reverse transcription quantitative real‐time polymerase chain reaction (RT‐qPCR) assay in peripheral blood mononuclear cells (PBMCs), with SYBR Green Master mix (BioFACT™ 2X Real‐Time PCR Master Mix, South Korea) in Bio‐Rad IQ5 system (Bio‐Rad, USA). Consequently, fold changes were calculated based on 2^−ΔΔCt^ method.

**TABLE 1:** The sequence of gene primers for RT‐qPCR.

AMPK	Forward TGATGACCATGTGCCAAC Reverse CCCTGATATCTTTGATTGTGG
AKT	Forward CTCTTTCCAGACCCACGACC Reverse ACAGGTGGAAGAACAGCTCG
PDK‐1	Forward AGATGAGTGACCGAGGAGGT Reverse CAAAACCAGCCAGAGGCACT
β‐Actin	Forward GGTGAAGGTGACAGCAGT Reverse TGGGGTGGCTTTTAGGAT

Abbreviations: AKT, protein kinase‐B; AMPK, adenosine monophosphate‐activated protein kinase; PDK‐1, phosphoinositide‐dependent kinase 1; RT‐qPCR, reverse transcription quantitative real‐time polymerase chain reaction.

### 2.3 Laboratory analysis

After overnight fasting, venous blood samples were drawn to separate the serum and then stored at −80°C. Pre‐ and post‐intervention, fasting glucose serum (FBS), was determined based on enzymatic–colorimetric methods using commercial kits (Pars Azmoon Co., Tehran, Iran). Hemoglobin A1C (HbA1c) in whole blood was determined using commercial kit (Pars Azmoon Company in Pars Azmoon, Iran) and autoanalyzer (Hitachi‐917, Tokyo, Japan), while the serum levels of insulin were quantified based on the enzyme‐linked immunosorbent assay (ELISA) technique and commercial kits (Monobind, Lake Forest, CA, USA).

In order to assess IR at baseline and after 8 weeks, IR‐related indices including homeostatic model assessment of insulin sensitivity (HOMA‐S), disposition index, quantitative insulin sensitivity check index (QUICKI), and fasting insulin resistance index (FIRI) were calculated based on serum fasting glucose and insulin levels as well as homeostatic model assessment of IR (HOMA‐IR) and homeostasis model assessment of β‐cell dysfunction (HOMA‐B) contents, according to the following formula (Al‐Hakeim & Abdulzahra, 2015; Chen et al., 2015):

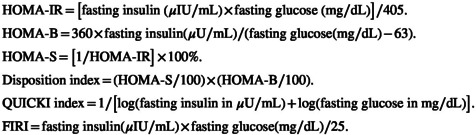




### 2.4 Primary and secondary outcomes

Changes in mRNA expression levels of AMPK, AKT, and PDK‐1 genes in PBMCs were considered the primary outcomes, while changes in IR‐related indices were defined as secondary endpoints.

### 2.5 Statistical analysis

Statistical analysis was performed using SPSS Statistics software ver. 23 (IBM SPSS Statistics, Armonk, USA) and GraphPad Prism (ver. 9). Kolmogorov–Smirnov test was done for checking the distribution of continuous variables, and data were expressed as mean (SD), while frequency and percent were documented for categorical variables. Both primary and secondary outcomes were done based on after‐treatment strategy. For inter‐ and intra‐comparison of continuous variables, independent sample *t*‐tests and paired sample *t*‐tests were applied, whereas for ordinal variables, Chi‐square and Sign tests were used. For controlling the covariates (i.e., baseline values and changes in energy intake) to compare at the end of intervention, the analysis of covariance (ANCOVA) test was done. The statistical significance level was defined as *p*‐value <.05.

## 3 RESULTS

**FIGURE 1:**
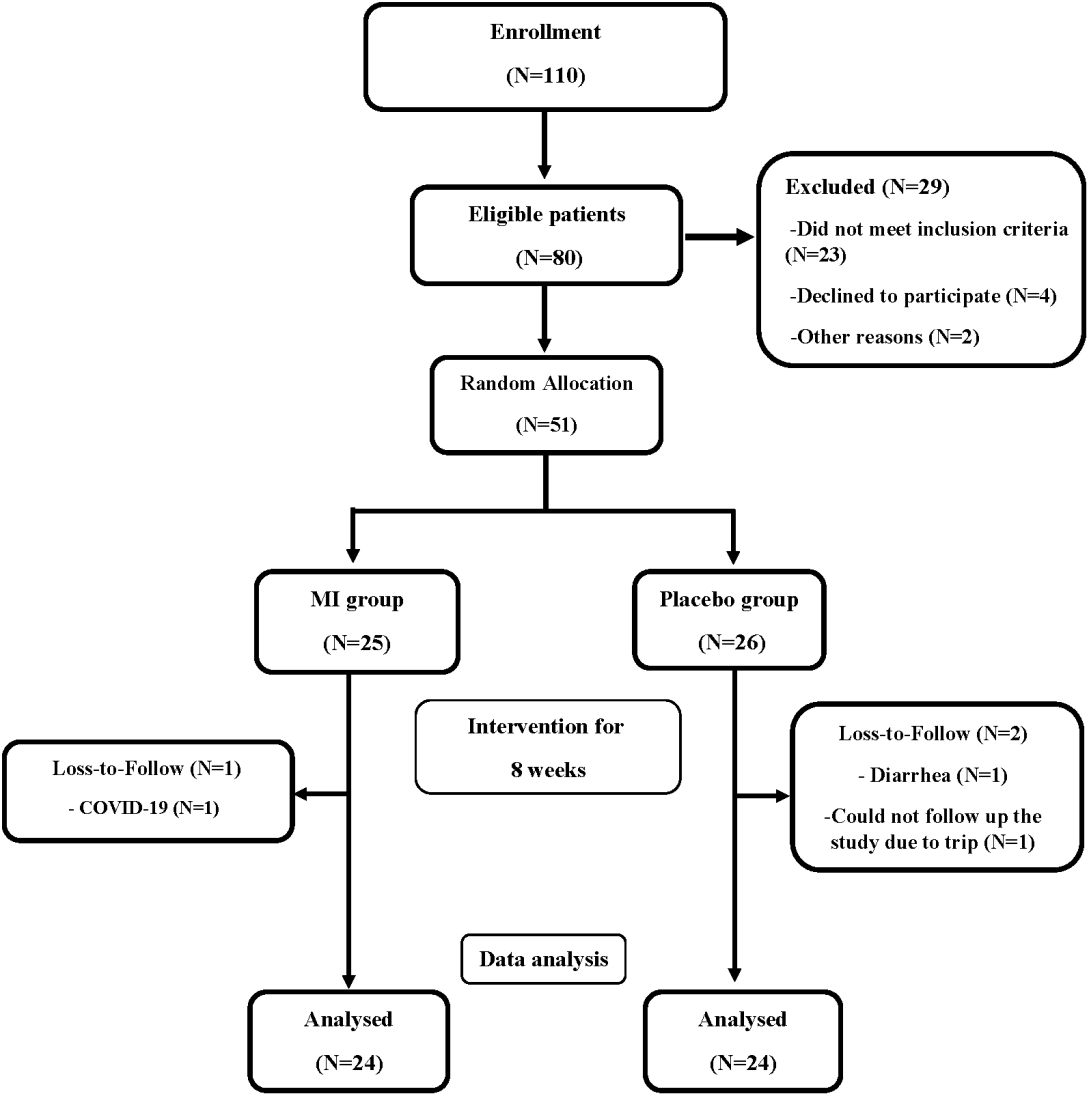
Trial profile.

Of totally 51 patients, three patients did not continue the study for reasons unrelated to the interventions (Figure 1).

Regarding the patients' characteristics at baseline, more than half of the patients were married with BMI between 30 and 35 Kg/m^2^ and suffered from grade II NAFLD. Apart from HOMA‐B, there were not any marked differences between the groups (Table 2).

**TABLE 2:** Patient's baseline characteristics.

Variables	MI (*N* = 24)	Placebo (*N* = 24)	*p* *
	Mean ± SD	Mean ± SD	
Age (years)	36.33 ± 8.49	37.50 ± 6.86	.603
FBS (mg/dL)	91.59 ± 8.58	97.52 ± 12.26	.059
Insulin (μIU/mL)	16.62 ± 7.79	13.99 ± 7.77	.248
HbA1c (%)	5.32 ± 0.46	5.41 ± 0.40	.465
HOMA‐IR	3.81 ± 2.00	3.42 ± 2.07	.503
HOMA‐B	219.45 ± 105.32	157.79 ± 80.47	**.027**

	%	%	
Male	54.2	47.6	.594
Married	83.3	87.5	.683
BMI status			0.686
<35 kg/m^2^	62.5	62.5	
35–39.9 kg/m^2^	37.5	37.5	
NAFLD severity			.558
Mild	37.5	45.8	
Moderate	62.5	54.2	

*Note*: Data presented as mean ± SD. Bold values indicate statistically significant *p* < .05.

Abbreviations: BMI, body mass index; FBS, fasting blood sugar; HbA1c, glycosylated hemoglobin A1c; HOMA‐B, homeostatic model assessment of β‐cell dysfunction; HOMA‐IR, homeostatic model assessment of insulin resistance; MI, myo‐inositol; NAFLD, non‐alcoholic fatty liver disease.

* *p*‐value for continuous variables using independent sample *t*‐test and for categorical variables using Chi‐square test. This table has been previously published (Arefhosseini, Roshanravan, Asghari, et al., 2023; Arefhosseini, Roshanravan, Tutunchi, et al., 2023).

Based on our previous reports, within‐ and between‐group differences in physical activity levels were not statistically significant (*p* = .819) (Arefhosseini, Roshanravan, Asghari, et al., 2023; Arefhosseini, Roshanravan, Tutunchi, et al., 2023). Marked reduction was observed in energy intake in both groups (−579.17 Kcal in MI arm vs. −688.65 Kcal in the placebo arm), while inter‐group changes were not statistically significant (*p* = .177) (Arefhosseini, Roshanravan, Asghari, et al., 2023; Arefhosseini, Roshanravan, Tutunchi, et al., 2023). Although the total dietary intake of macronutrients (inc. carbohydrates, protein, and fat) as well as saturated fatty acids reduced significantly in both groups, inter‐group differences did not reach statistically significant levels. The intake of anti‐oxidant micronutrients was not significantly different between the two groups at the end of the study (Arefhosseini, Roshanravan, Asghari, et al., 2023; Arefhosseini, Roshanravan, Tutunchi, et al., 2023).

Furthermore, we previously reported the anti‐obesity features of MI supplementation, that is, significant decreases in body weight (−4.72 Kg in MI group vs. −3.27 Kg in the placebo group, *p* = .049), after adjusting for the potential confounders (Arefhosseini, Roshanravan, Asghari, et al., 2023; Arefhosseini, Roshanravan, Tutunchi, et al., 2023). Despite the significant intra‐group reductions in BMI in both studied groups, changes were higher in the MI group (−1.66 Kg/m^2^ in MI group vs. −1.16 Kg/m^2^ in the placebo group, *p* = .052) (Arefhosseini, Roshanravan, Asghari, et al., 2023; Arefhosseini, Roshanravan, Tutunchi, et al., 2023).

**FIGURE 2:**
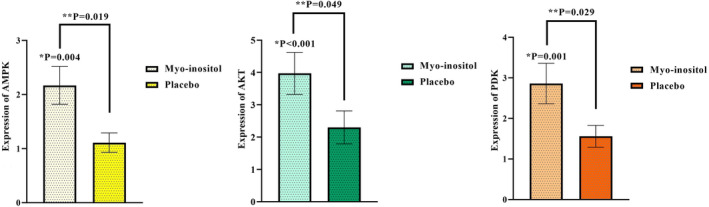
Result of intervention. Fold change of AMPK, AKT, and PDK‐1 mRNAs expression. Values are the mean of fold change ± SEM. Data analysis was done using the one sample *t*‐test and ANCOVA. *p < .05 versus baseline. **p < .05 versus Placebo. *p* < .05 statistically significant. AMPK, adenosine monophosphate‐activated protein kinase; AKT, protein kinase‐B; PDK‐1, phosphoinositide‐dependent kinase 1.

Changes in mRNA expression levels of AMPK/PI3K/AKT pathway genes are shown in Figure 2. Compared to the steady‐state expression, using one sample *t*‐test, MI group showed significant increased fold changes of AMPK, AKT, and PDK‐1 (*p* = .004, *p* < .001, and *p* = .001, respectively), while placebo group could not reach to statistical significant levels (Figure 2). Post‐intervention, the fold change of AMPK, AKT, and PDK‐1 was approximately two times higher in MI group than placebo group after controlling for the confounders [fold change of AMPK: 2.17 ± 0.35 (MI group) vs. 1.11 ± 0.18 (placebo group); fold change of AKT: 3.97 ± 0.65 (MI group) vs. 2.30 ± 0.51 (placebo group); fold change of PDK‐1: 2.86 ± 0.50 (MI group) vs. 1.56 ± 0.27 (placebo group) (*p* = .019, *p* = .049, and *p* = .029, respectively)] (Figure 2).

We previously reported the beneficial effect of MI supplementation on IR (Arefhosseini, Roshanravan, Tutunchi, et al., 2023). In this study, apart from QUICKI index, all IR indices improved in MI group (*p* < .05), while no significant differences in the indices were found in the placebo group (Table 3). By controlling the confounders, MI group showed statistically significant improvements in all IR indices, except QUICKI (Table 3).

**TABLE 3:** Changes in insulin resistance indices from baseline to week 8.

Variables	MI (*n* = 24)	Placebo (*n* = 24)	*p*
	Mean ± SD	Mean ± SD	
HOMA‐S			
Baseline	32.5 ± 16.3	40.6 ± 26.6	.149**
End	45.6 ± 26.5	36.1 ± 13.3	
MD (95% CI)	−13.1 (−23.2, −3.11)	4.6 (−6.6, 15.7)	.044***
*p* *	**.007**	.977	
QUICKI index			
Baseline	2.8 ± 0.1	3.0 ± 0.3	.047**
End	2.9 ± 0.2	2.9 ± 0.1	
MD (95% CI)	−0.09 (−0.19, 0.01)	0.05 (−0.08, 0.18)	.865***
*p* *	.065	.932	
Glu/Ins			
Baseline	6.5 ± 2.6	9.3 ± 6.9	.386^††^
End	8.3 ± 4.4	8.1 ± 2.7	
MD (95% CI)	−1.8 (−3.6, 0.03)	1.2 (−1.9, 4.3)	**.043** ***
*p* ^†^	**.024**	.864	
Deposition index			
Baseline	6318.8 ± 2583.7	5386.0 ± 3082.6	.149^††^
End	7610.1 ± 2069.0	4973.0 ± 1890.1	
MD (95% CI)	−1291 (−2383.0, −199.5)	412.9 (−663.2, 1489.1)	<.001***
*p* ^†^	**.018**	.927	
FIRI			
Baseline	61.8 ± 32.5	55.3 ± 33.5	.149^††^
End	43.7 ± 17.4	50.6 ± 17.5	
MD (95% CI)	18.1 (5.4, 30.7)	4.7 (−11.3, 20.8)	.043***
*p* ^†^	**.005**	.732	

*Note*: Mean (SD) and mean difference (95% CI) are presented for data. Bold values indicate statistically significant *p* < .05.

Abbreviations: FIRI, fasting insulin resistance index; Glu/Ins, glucose‐to‐insulin ratio; HOMA‐S, homeostatic model assessment of insulin sensitivity; MI, myo‐inositol; QUICKI index, quantitative insulin sensitivity check index.

* *p*‐value for paired sample *t*‐test;

** *p*‐value for independent sample *t*‐test;

*** *p*‐value for ANCOVA test (adjusted for baseline values and energy intake);

^†^ *p*‐value for related samples Wilcoxon signed rank test;

^††^ *p*‐value for Mann–Whitney *U* test.

## 4 DISCUSSION

**FIGURE 3:**
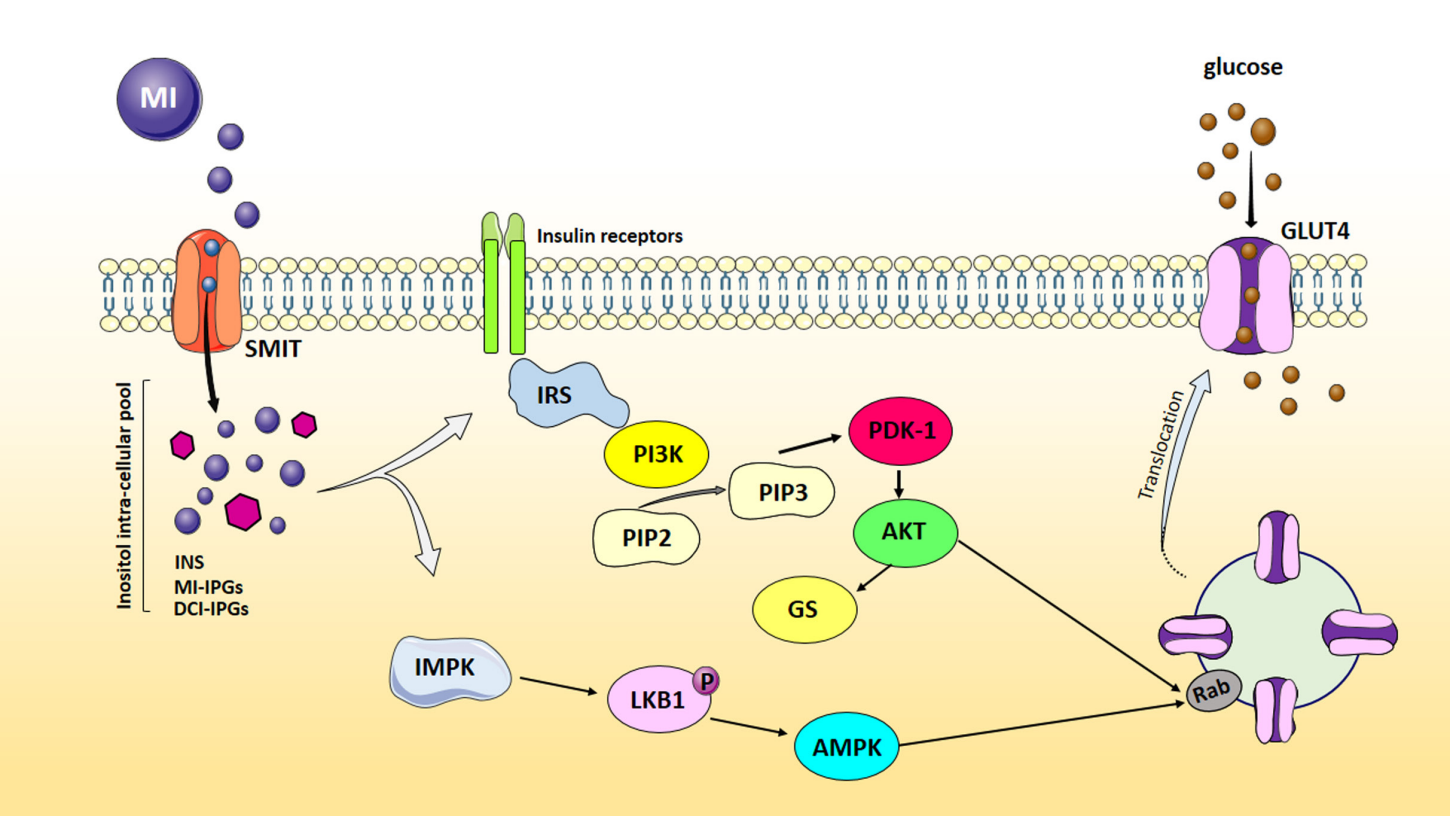
Possible triggered molecular pathways by MI supplementation, leading to the treatment of IR in patients with NAFLD. MI participates in intra‐cellular pool of phosphatidylinositols and activates IPMK which phosphorylates LKB1 and thereby upregulates AMPK. On the other side, increased generation of MI‐IPGs and DCI–IPGs directly upregulate IRS, PDK‐1, and AKT. AKT activation results in the phosphorylation of Rab and facilitating the translocation of GLUT‐4 and lowering blood glucose levels (S12). AKT stimulates glucose synthase (GS) activity, resulting in reduced hepatic glucose synthesis. AKT, protein kinase‐B; AMPK, adenosine monophosphate‐activated protein kinase; DCI‐IPGs, d‐chiro inositol containing inositol phosphoglycans; GLUT‐4 glucose transporter 4; GS, glycogen synthase; IMPK, inositol phosphate multikinase; INS, inositols; MI, myo‐inositol; MI‐IPGs, Myo‐inositol containing inositol phosphoglycans; PDK‐1 phosphoinositide‐dependent kinase 1; PI3K, phosphoinositide 3 kinase IRS insulin receptor substrates; PIP2, Phosphatidylinositol‐4,5‐bisphphosphate; PIP3, phosphatidylinositol‐3,4,5‐triphphosphate; SMIT, sodium/myo‐inositol transporter.

To the current body of evidence, our study seems to be the first RCT assessing the influence of MI supplementation on IR through AMPK/PI3K/AKT signaling pathway in obese patients with NAFLD. IR is considered a cause, an outcome, or even an epiphenomenon of NAFLD (Arefhosseini et al., 2022). Therefore, targeting IR in the treatment of NAFLD seems to be useful (Arefhosseini et al., 2022). In the milieu of metabolic‐endocrine disease, AMPK/PI3K/AKT pathway regulates the function of a plethora of downstream molecular mediators involved in cellular homeostasis, particularly in insulin‐sensitive tissues and plays role in the function of β‐cell (Perez‐Frances et al., 2021; Savova et al., 2023). AMPK, AKT, and PDK‐1—the main involved genes in the aforementioned pathway—activate the regulatory proteins responsible for glucose and lipid metabolism and are widely implemented in the treatment of T2DM and NAFLD (Cabrera‐Cruz et al., 2020; El‐Masry et al., 2015; Savova et al., 2023; Zhang et al., 2019). AMPK as a known metabolic regulator catalyzes ATP production, increases lipid oxidation, and suppresses de novo lipogenesis as well as oxidative‐inflammatory pathways and results in improved insulin sensitivity (Garcia & Shaw, 2017; Zhou et al., 2023). Moreover, AMPK activators are considered potent anti‐diabetic agents (Zhou et al., 2023). Our result demonstrated that daily supplementation with 4 g of MI markedly enhanced the mRNA expression levels of AMPK in PBMCs, approximately two times more than the placebo group (Figure 2). These findings are in accordance with the results of most cellular and experimental studies (Bu et al., 2022; Cabrera‐Cruz et al., 2020; Luo et al., 2022). For example, Cabrera‐Cruz et al. (2020), in a cell‐line study on PCOS featured‐human endometrial cells, documented the significant activation of AMPK through sodium/myo‐inositol transporter 1 with subsequent increased protein levels of glucose transporter‐4 (GLUT‐4) after MI supplementation. The activation of AMPK pathway resulted in improved glucose uptake and IR. The positive effects of MI supplementation on hepato‐pancreatic mRNA expression levels of AMPK in crabs and fishes went under high‐fat diet (HFD) were also reported (Bu et al., 2022; Luo et al., 2022). Increased AMPK expression led to a significant improvement in lipid metabolism and anti‐oxidant activities in the mentioned studies. Indeed, similar findings were shown by Yang et al. (2022) which 12 weeks of d‐chiro‐inositol (DCI) (50 and 100 mg/Kg/day, respectively) treatment in mice fed with HFD, attenuated obesity, liver fat accumulation, and glycemic profile (e.g., FBS and insulin levels) by increasing hepatic protein expression levels of phosphorylated‐AMPKα at Thr172. Despite the lack of human studies, our findings are in agreement with the latter. Although the exact effect of INSs on AMPK has not fully been understood, the underlying mechanism of action should be explained by the influx of MI molecules in the intra‐cellular pool of phosphatidylinositol including IP3, PIP, PIP2, and PIP3 (Bizzarri et al., 2017; Cabrera‐Cruz et al., 2020). These changes simultaneously trigger AMPK and PI3K/AKT pathways and are followed by the subsequent changes in inositol phosphate multikinase (IPMK) activity (Bizzarri et al., 2017). IMPK phosphorylates the serine–threonine kinase, LKB1, and thereby activates AMPK, while, in turn, AMPK upregulates GLUT‐4 protein expression through G‐protein phosphorylation and exerts glucose‐lipid‐lowering features (Bizzarri et al., 2017; Cabrera‐Cruz et al., 2020) (Figure 3). Furthermore, PI3K/AKT activation, namely through activated AKT and PDK‐1, directly mediates glucose homeostasis and insulin signaling (Kuşcu et al., 2016). We found that MI supplementation obviously enhanced AKT and PDK‐1 expression in PBMCs (Figure 2). To date, some experimental studies showed insulin‐mimetic effects of INSs throughout PI3K/AKT pathway regulation. Kuşcu et al. (2016) exposed blastocyst‐stage embryo to MI, in order to assess the post‐implantation development in pseudopregnant female mice. In this in vitro model, MI exposure led to the activation of AKT (Kuşcu et al., 2016). Following them, an animal study examined the effects of chronic administration of MI (0.9 or 1.2 mg/g/day, orally or intraperitoneally) and reported that MI treatment increased PI3K/AKT phosphorylation in skeletal muscle after 15 days (Croze et al., 2013). Alleviated glucose tolerance was related to improved insulin sensitivity (Croze et al., 2013). According to the evidence, INSs (especially DCI and MI) are natural compounds exhibiting the same glucose‐lowering effects with medical treatments like metformin, besides minimum side effects compared with them (Cheng et al., 2019). Cheng et al. (2019) showed greater effects of intra‐gastric DCI (50 mg/Kg/day) compared with metformin (200 mg/Kg/day) administration on mice with obesity after 8 weeks. DCI administration decreased FBS, improved glucose intolerance through PI3K/AKT pathway, namely by upregulating insulin receptor substrate (IRS) and PI3Kp85 as well as IRS‐2 and AKT phosphorylation (Cheng et al., 2019). Moreover, suppressed hepatic vacuolization and steatosis were evident after DCI load in HepG2 cells (Cheng et al., 2019). Robust evidence has highlighted the fine‐tuned PI3K/AKT axis through MI administration, as the participation of MI in phosphoinositide cell cycle increases the generation of MI and DCI—containing inositol glycans (IPG) which, in turn, suppress adenylate cyclase and cAMP kinase, exert insulin‐like functions, regulate glucose metabolism, and directly upregulate IRS and AKT (Bizzarri et al., 2017). As shown in Figure 3, AKT activation results in two main effects including the translocation and expression of GLUT‐4 in cell surface throughout the activation/phosphorylation of G‐proteins such as Rab and subsequently, upregulated GLUT‐4 results in lowered glucose levels (Lankatillake et al., 2019). Increased AKT mRNA expression also deactivates glycogen synthase kinase 3 (GSK3) which promotes glucose synthase activity leading to reduced hepatic glucose synthesis (Lankatillake et al., 2019). An alternative mechanism of MI appears to be explained by the downregulation of cytosolic phosphoenolpyruvate carboxykinase and glucose‐6‐phosphatase involved in gluconeogenesis through PKCε‐IRS/PI3K/AKT axis activation (Cheng et al., 2019; Croze et al., 2013).

To shed light on the clinical manifestation of the mentioned molecular changes, IR‐related indices were assessed as well. Our results showed that an 8‐week MI supplementation considerably improved glycemic response and IR in patients with NAFLD (Arefhosseini, Roshanravan, Tutunchi, et al., 2023). Adding strength to the current knowledge, results of the present trial revealed a remarkable reduction in IR indices (Table 3). In this regard, marked increases in HOMA‐S and deposition index as the indicators of insulin sensitivity as well as significant reduction in FIRI represent the anti‐hyperglycemic feature of MI (Table 3). These results are similar to the findings of animal (Antony et al., 2017) and human studies (Kim et al., 2005), in patients with PCOS and T2DM (Sung et al., 2012). A meta‐analysis by Miñambres et al. (Miñambres et al., 2019) (on 20 RCTs) with various kinds of IR‐related disorders reported reduced levels of glucose, insulin, and HOMA‐IR after supplementation with INSs. In PCOS, supplementation with MI significantly reduced HOMA‐IR, metabolic, and hormonal factors (Kamenov & Gateva, 2020). Moreover, improved IR state after MI supplementation was even greater than metformin and oral contraceptive treatments with fewer side effects in patients with PCOS (Facchinetti et al., 2019). Similar results were also reported in patients with gestational diabetes (Swathi et al., 2021). These findings have been established by Unfer et al. (2017) in a meta‐analysis (including nine RCTs) which assessed the effects of MI supplementation for 12–24 weeks on PCOS. The suggested mechanism could be attributed to its insulin‐sensitizing effect apart from weight reduction effects (Kim et al., 2005). Moreover, MI supplementation improves glucose uptake and transit time as well as ameliorating adipocyte insulin stimulation and suppressing hepatic gluconeogenesis (Cheng et al., 2019; Croze et al., 2013).

To the best of my knowledge, the present RCT appears to be the first RCT assessing the anti‐hyperglycemic effects of MI through AMPK/PI3K/AKT pathway in obese patients with NAFLD. A most important strength of our study was the intervention on newly diagnosed patients with NAFLD, evaluating the molecular pathway involved in IR by RT‐qPCR as well as frequent visits and high compliance rate. Our study had some limitations including short duration and administrating relatively low doses of MI. In addition, self‐reported physical activity and dietary intake could influence our findings.

## 5 CONCLUSION

In conclusion, MI supplementation (4 g/day) for 8 weeks along with dietary recommendations, upregulated AMPK, AKT, and PDK‐1 mRNA expression in PBMCs and manifested as improved IR indices in patients with NAFLD.

## AUTHOR CONTRIBUTIONS

**Taha Aghajani:** Conceptualization (equal); data curation (equal); investigation (equal); writing – original draft (equal). **Sara Arefhosseini:** Data curation (equal); investigation (equal); writing – original draft (equal). **Mehrangiz Ebrahimi‐Mameghani:** Conceptualization (equal); data curation (equal); writing – review and editing (equal). **Reza Safaralizadeh:** Conceptualization (equal); funding acquisition (equal); resources (equal); supervision (equal).

## FUNDING INFORMATION

This study was funded by the ‘Research Vice‐Chancellor’ of Tabriz University of Medical Sciences, Tabriz, Iran.

## CONFLICT OF INTEREST STATEMENT

The authors declare that they have no competing interests.

## ETHICS STATEMENT

The protocol of the present clinical trial was in accordance with the standards of the Ethics Committee of Tabriz University of Medical Sciences. In this study, an informed consent form was confirmed by the ethical committee of Tabriz University of Medical Sciences (Ethics code: TBZMED. REC.1400.567).

## Data Availability

The datasets used and/or analyzed in this study are available from the corresponding author on a reasonable request.
